# Supplementary material for: LTR Retroelements and Bird Adaptation to Arid Environments
Source: Int J Mol Sci. 2023 Mar 28;24(7):6332. doi: 10.3390/ijms24076332 (PMC10094322; doi:10.3390/ijms24076332)
Supplement: Supplementary file 1 [file ijms-24-06332-s001.zip › ijms-2211538-SM.pdf]

Supplementary materials

LTR retroelements and bird adaptation to arid environments.

Elisa Carotti[1†], Edith Tittarelli[1†], Adriana Canapa[1], Maria Assunta Biscotti[1], Federica Carducci[1*] and Marco Barucca[1].

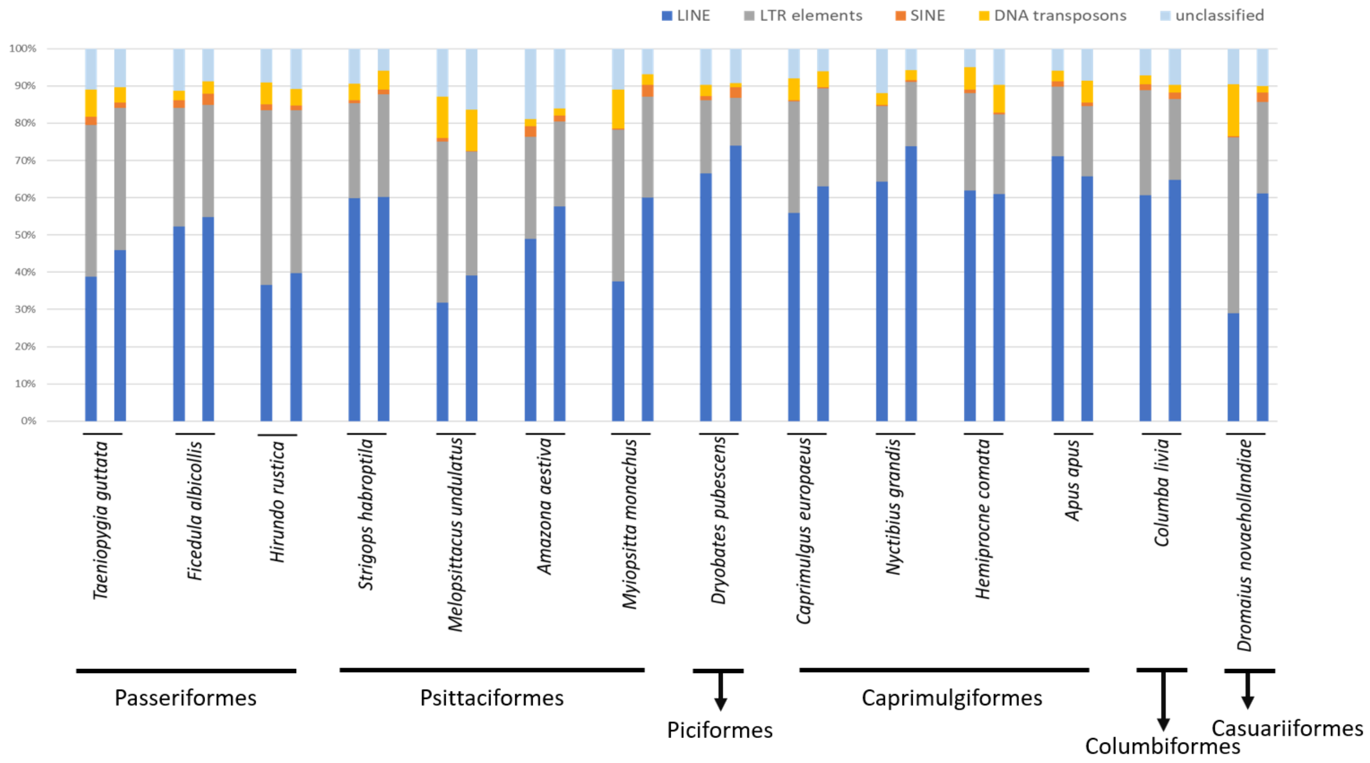

Supplementary Figure S1. TE relative abundance obtained analyzing 14 bird species. For each species the column on the left represents the values obtained considering chromosome level assembly while the column on the right represents the values obtained considering scaffold level assembly.

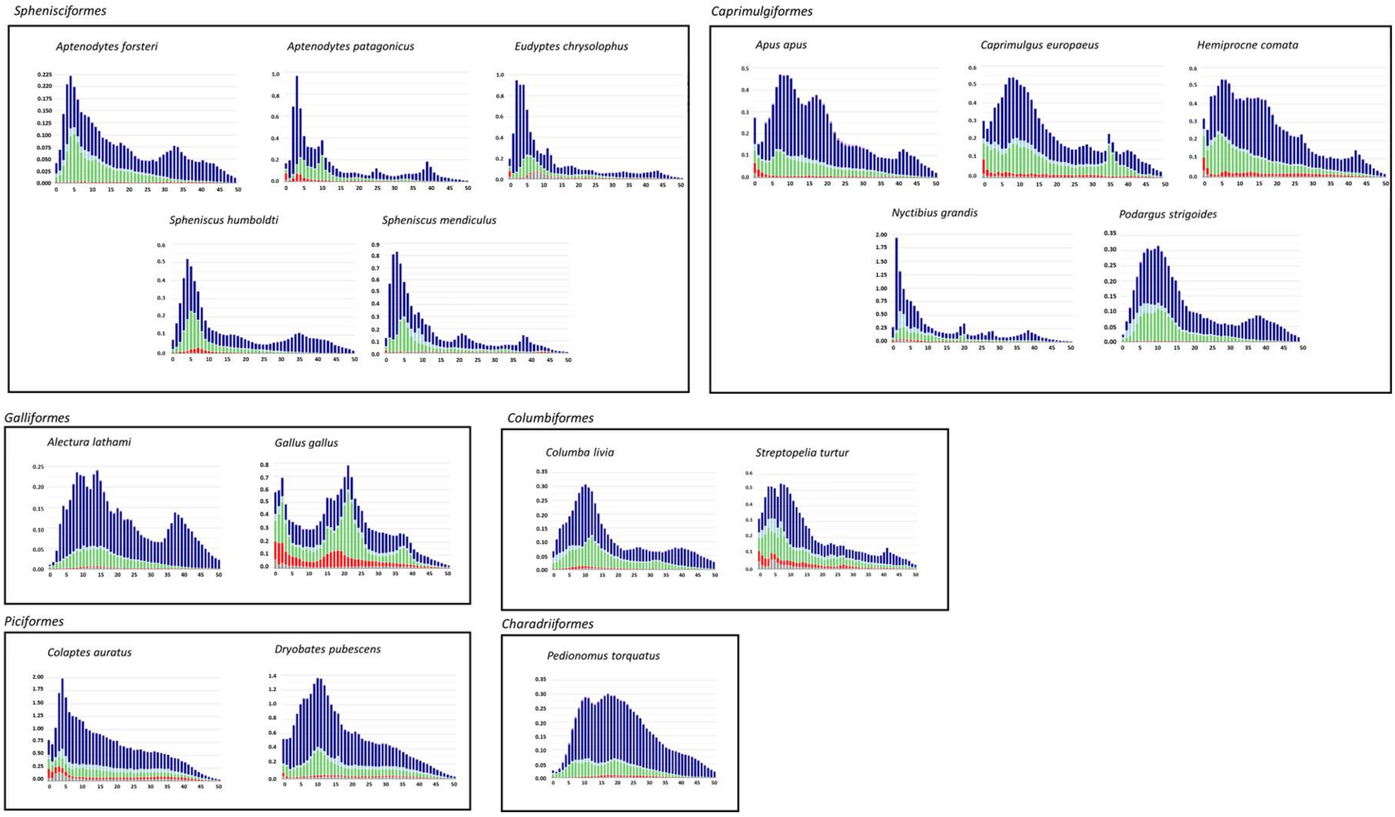

Supplementary Figure S2. TE landscape plots obtained by Kimura distance-based copy divergence analyses of some species belonging to Sphenisciformes, Caprimulgiformes, Galliformes, Columbiformes, Piciformes, and Charadriiformes. X axis: Kimura substitution level (CpG adjusted); Y axis: percent of genome.

Supplementary Table S1. Accession numbers and information regarding the assembly level of genomes belonging to bird species used in this study are reported. For each species the common name is listed in the right column.

Assembly accession	Assembly level	Species	Common Name
**GCA_013399715.1**	Scaffold	*Alectura lathami*	Australian brush-turkey
**GCA_017639355.1**	Chromosome	*Amazona aestiva*	Blue-fronted amazon
**GCA_001420675.1**	Scaffold		
**GCF_000699145.1**	Scaffold	*Aptenodytes forsteri*	Emperor penguin
**GCA_010087175.1**	Scaffold	*Aptenodytes patagonicus*	King penguin
**GCA_020740795.1**	Chromosome	*Apus apus*	Common swift
**GCA_020740585.1**	Scaffold		
**GCA_907165065.1**	Chromosome	*Caprimulgus europaeus*	Eurasian nightjar
**GCA_907165095**	Scaffold		
**GCA_013396415.1**	Scaffold	*Casuarius casuarius*	Southern cassowary
**GCA_015227895.2**	Chromosome	*Colaptes auratus*	Northern flicker
**GCA_001887795.1**	Chromosome	*Columba livia*	rock pigeon
**GCF_000337935.1**	Scaffold		
**GCA_016128335.1**	Chromosome	*Dromaius novaehollandiae*	Emu
**GCA_013396795.1**	Scaffold		
**GCA_014839835.1**	Chromosome	*Dryobates pubescens*	Downy woodpecker
**GCF_000699005.1**	Scaffold		
**GCA_010084205.1**	Scaffold	*Eudyptes chrysolophus*	Macaroni penguin
**GCF_000247815.1**	Chromosome	*Ficedula albicollis*	Collared flycatcher
**GCF_000247815.1**	Scaffold		
**GCA_000002315.5**	Chromosome	*Gallus gallus*	Chicken
**GCA_020745705.1**	Chromosome	*Hemiprocne comata*	Whiskered treeswift
**GCA_020745695.1**	Scaffold		
**GCA_015227805.3**	Chromosome	*Hirundo rustica*	Barn swallow
**GCA_015227815.3**	Scaffold		
**GCF_012275295.1**	Chromosome	*Melopsittacus undulatus*	Budgerigar
**GCA_012275275.1**	Scaffold		
**GCA_017639245.1**	Chromosome	*Myiopsitta monachus*	Monk parakeet

**GCA_013036005.1**	Scaffold		
**GCA_013368605.1**	Chromosome	*Nyctibius grandis*	Great potoo
**GCA_013368595.1**	Scaffold		
**GCA_001700915.1**	Chromosome	*Passer domesticus*	House sparrow
**GCA_013398155.1**	Scaffold	*Pedionomus torquatus*	Plains-wanderer
**GCA_013399755.1**	Scaffold	*Podargus strigoides*	Tawny frogmouth
**GCA_003343005.1**	Scaffold	*Rhea americana*	Greater rhea
**GCA_010076325.1**	Scaffold	*Spheniscus humboldti*	Humboldt's penguin
**GCA_010077915.1**	Scaffold	*Spheniscus mendiculus*	Galapagos penguin
**GCA_901699155.2**	Chromosome	*Streptopelia turtur*	European turtle-dove
**GCF_004027225.2**	Chromosome	*Strigops habroptila*	Kākāpō
**GCA_004011185.1**	Scaffold (partial)		
**GCF_000698965.1**	Scaffold	*Struthio camelus australis*	Common ostrich
**GCF_003957565.2**	Chromosome	*Taeniopygia guttata*	Zebra finch
**GCA_003957525.2**	Scaffold		

Supplementary Table S2. Accession numbers of scaffold-level assemblies.

Species	Assembly accession	Assembly Level
H. rustica	GCA_015227815.3	scaffold
T. guttata	GCA_003957525.2	scaffold
F. albicollis	GCA_000247815.1	scaffold
M. undulatus	GCA_012275275.1	scaffold
S. habroptila	GCA_004011185.1	scaffold
A. aestiva	GCA_001420675.1	scaffold
M. monachus	GCA_013036005.1	scaffold
D. pubescens	GCF_000699005.1	scaffold
C. livia	GCF_000337935.1	scaffold
N. grandis	GCA_013368595.1	scaffold
C. europaeus	GCA_907165095	scaffold

H. comata	GCA_020745695.1	scaffold
A. apus	GCA_020740585.1	scaffold
D. novaehollandiae	GCA_013396795.1	scaffold

Supplementary Table S3. Accession numbers of complete mitochondrial genome and positions related to 16S rDNA sequence. These sequences were used in vegan analyses. Species considered were listed and their abbreviations were reported in brackets.

Species	Accession number – mitochondrial DNA 16s
A. aestiva (Aae)	NC_033336.1:1106-2673
A. apus (Aap)	NC_008540.1:1115-2716
A. forsteri (Afo)	NC_027938.1:1119-2704
A. lathami (Ala)	MW574353.1:2248-3860
A. patagonicus (Apa)	NC_045377.1:1140-2692
C. casuarius (Cca)	MN356153.1:1107-2701
C. auratus (Cau)	JAAWVA010000182.1:20328-21925
C. livia (Cli)	GU908131.1:1115-2700
D. novaehollandiae (Dno)	NC_002784.1:1108-2703
D. pubescens (Dpu)	MN356145.1:1114-2707
E. crhysolophus (Ecr)	MW074963.1:1117-2707
F. albicollis (Fal)	NC_021621.1:1120-2715
G. gallus (Gga)	KX987152.1:2351-3972
H. comata (Hco)	MN356189.1:1116-2705
H. rustica (Hru)	MN356225.1:1112-2719
M. monachus (Mmo)	NC_027844.1:2364-3942
M. undulatus (Mun)	EF450826.1:1117-2688
N. grandis (Ngr)	MN356216.1:1121-2718
P. domesticus (Pdo)	MN356394.1:1116-2715
P. strigoides (Pst)	MW883530.1:1100-2713
P. torquatus (Pto)	MN356368.1:1113-2696
R. americana (Ram)	Y16884.3:1103-2685
S. camelus australis (Sca)	NC_002785.1:1104-2683
S. habroptila (Sha)	MZ128785.1:1085-2674
S. humboldti (Shu)	MK760995.1:1145-2705
S. mendiculus (Sme)	MK760994.1:1145-2704
S. turtur (Stu)	CABFKC020000024.1:41166-42762
T. guttata (Tgu)	DQ453515.1:1120-2713

Supplementary Table S4. P-distance matrix performed on 16S rDNA sequences of species considered in the present study, exception made for *Caprimulgus europaeus* (please see material and methods for further details). Abbreviations used for species names were explained in Supplementary Table S3.

	Hru	Pdo	Tgu	Fal	Sha	Aae	Mmo	Mun	Dpu	Cau	Afo	Apa	Shu	Sme	Ech	Hco	Aap	Pto	Ngr	Cli	Stu	Dno	Cca	Sca	Ram	Pst	Ala	Gga
Hru																												
Pdo	0.10																											
Tgu	0.10	0.06																										
Fal	0.10	0.08	0.08																									
Sha	0.20	0.20	0.19	0.20																								
Aae	0.22	0.22	0.21	0.21	0.15																							
Mmo	0.21	0.20	0.20	0.19	0.13	0.12																						
Mun	0.21	0.21	0.20	0.20	0.15	0.14	0.13																					
Dpu	0.21	0.20	0.21	0.20	0.20	0.20	0.20	0.19																				
Cau	0.21	0.20	0.20	0.21	0.20	0.21	0.21	0.21	0.13																			
Afo	0.18	0.17	0.18	0.18	0.18	0.20	0.18	0.18	0.18	0.18																		
Apa	0.18	0.17	0.18	0.18	0.18	0.20	0.18	0.17	0.18	0.17	0.02																	
Shu	0.18	0.17	0.18	0.17	0.17	0.20	0.17	0.17	0.17	0.18	0.08	0.08																
Sme	0.18	0.17	0.18	0.18	0.17	0.20	0.17	0.17	0.17	0.18	0.08	0.08	0.00															
Ech	0.18	0.17	0.17	0.17	0.18	0.20	0.18	0.17	0.17	0.17	0.08	0.08	0.06	0.06														
Hco	0.17	0.17	0.17	0.17	0.17	0.20	0.18	0.18	0.18	0.18	0.15	0.16	0.15	0.15	0.15													
Aap	0.18	0.19	0.18	0.18	0.17	0.19	0.17	0.18	0.17	0.18	0.14	0.15	0.13	0.13	0.15	0.12												
Pto	0.19	0.19	0.19	0.19	0.19	0.20	0.19	0.18	0.19	0.19	0.16	0.15	0.16	0.16	0.15	0.16	0.17											
Ngr	0.21	0.20	0.20	0.19	0.18	0.21	0.19	0.19	0.19	0.20	0.17	0.17	0.16	0.16	0.16	0.16	0.17	0.18										
Cli	0.19	0.19	0.18	0.19	0.19	0.19	0.19	0.19	0.19	0.20	0.16	0.16	0.16	0.16	0.16	0.17	0.17	0.17	0.18									
Stu	0.19	0.18	0.18	0.18	0.19	0.20	0.20	0.19	0.18	0.20	0.17	0.16	0.16	0.16	0.16	0.17	0.17	0.17	0.18	0.08								
Dno	0.20	0.19	0.18	0.19	0.20	0.20	0.20	0.20	0.20	0.20	0.18	0.17	0.17	0.17	0.16	0.19	0.19	0.18	0.20	0.18	0.19							
Cca	0.20	0.20	0.19	0.19	0.20	0.21	0.20	0.20	0.20	0.21	0.16	0.15	0.16	0.16	0.15	0.19	0.19	0.17	0.19	0.18	0.19	0.09						
Sca	0.20	0.20	0.19	0.20	0.19	0.21	0.20	0.19	0.20	0.20	0.16	0.15	0.14	0.15	0.15	0.19	0.18	0.17	0.18	0.19	0.19	0.13	0.13					
Ram	0.18	0.19	0.18	0.18	0.20	0.21	0.20	0.19	0.19	0.20	0.17	0.17	0.17	0.17	0.17	0.18	0.17	0.18	0.19	0.20	0.19	0.14	0.14	0.14				
Pst	0.22	0.21	0.22	0.22	0.21	0.21	0.21	0.21	0.18	0.20	0.18	0.18	0.19	0.19	0.19	0.19	0.18	0.19	0.20	0.19	0.20	0.20	0.20	0.21	0.21			
Ala	0.21	0.21	0.21	0.20	0.20	0.20	0.20	0.19	0.20	0.20	0.18	0.18	0.18	0.18	0.17	0.19	0.18	0.19	0.20	0.21	0.20	0.19	0.19	0.18	0.19	0.22		
Gga	0.21	0.20	0.21	0.20	0.22	0.22	0.22	0.21	0.22	0.21	0.19	0.18	0.19	0.19	0.18	0.20	0.19	0.19	0.21	0.20	0.20	0.20	0.20	0.19	0.21	0.22	0.18	
